# Atrial fibrillation in stroke patients: facts, numbers and current challenges

**DOI:** 10.1186/s42466-026-00538-9

**Published:** 2026-10-19

**Authors:** Katrin Wasser, Deborah Bochert, Johanna Rosemarie Leyhe, Marianne Hahn, Sonja Gröschel, Klaus Gröschel, Rolf Wachter

**Affiliations:** 1 Department of Neurology, University of Göttingen Medical Centre, Göttingen, Germany; 2 Department of Neurology, University Medical Centre of the Johannes Gutenberg University Mainz, Mainz, Germany; 3 Department of Cardiology, University Hospital Leipzig, Liebigstraße 20, Haus 4, Leipzig, 04103 Germany; 4 Partner site Göttingen, German Centre for Cardiovascular Research (DZHK), Göttingen, Germany

**Keywords:** Atrial fibrillation, Ischaemic stroke, Intracranial haemorrhage, ECG monitoring, Anticoagulation

## Abstract

Atrial fibrillation (AF) is a leading, modifiable risk factor for ischaemic stroke, with 20–30% of stroke patients harbouring underlying AF. Management of AF, however, differs substantially depending on clinical context. In ischaemic stroke patients with pre-known AF, direct oral anticoagulants (DOACs) are the cornerstone of secondary prevention, with initiation timing guided by infarct severity following a risk-adapted approach. In patients with no history of AF, prolonged cardiac rhythm monitoring after stroke or transient ischaemic attack improves AF detection rates. However, it remains uncertain whether this translates into a reduced risk of recurrent stroke, with randomised trials such as Find-AF 2 ongoing. Subclinical AF, detected via implantable devices, is associated with an increased stroke risk correlating with arrhythmia burden; recent trials (ARTESiA, NOAH-AFNET 6) suggest that anticoagulation may reduce ischaemic stroke risk in this population, but at the cost of increased bleeding, necessitating individualised decision-making. Particularly challenging are anticoagulation decisions with concomitant cerebral amyloid angiopathy or prior intracerebral haemorrhage (ICH) due to competing risks of thromboembolism and haemorrhage. Recent meta-analyses indicate that resuming DOACs after ICH reduces ischaemic events but significantly raises the risk of recurrent haemorrhage, without clear improvement in net clinical benefit. New treatment options, such as left atrial appendage closure, carotid artery filter systems, factor XIa inhibitors or rhythm control, must demonstrate in adequately powered randomised controlled trials that they can optimise treatment outcomes in this complex and growing patient group. This article summarises some common interdisciplinary neurological and cardiological questions and discusses the most recent evidence on AF-related stroke prevention across various patient groups, highlighting the importance of individualised, multidisciplinary, and imaging-informed decision-making.

## Background

Atrial fibrillation (AF) is the most prevalent cardiac arrhythmia and is considered a significant risk factor for ischaemic stroke [1]. Recent studies indicate that up to 20–30% of patients who experience an ischaemic stroke have underlying AF [2–4]. AF-related strokes are more severe and associated with increased mortality and recurrence rates, especially in the absence of adequate anticoagulation therapy [5].

The incidence of AF increases with age [6] and is often associated with comorbidities such as hypertension, diabetes mellitus, and heart failure [7] and will therefore become even more important in ageing societies.

Recent research emphasises the importance of early identification of AF in stroke patients, as timely diagnosis can optimise anticoagulation therapy and potentially improve clinical outcomes [8].

A relatively new entity is subclinical AF, which is detected by implanted devices in patients without prior clinical diagnosis of AF and in the absence of symptoms attributable to the arrhythmia. This article aims to summarise the current evidence on the relationship between AF and ischaemic or haemorrhagic stroke and discuss the challenges in diagnosis and management. It highlights the significance of an interdisciplinary approach to enhance patient care in three key patient groups: (1) ischaemic stroke patients with pre-existing AF with or without oral anticoagulation, (2) ischaemic stroke patients with clinical AF detected after stroke, and (3) ischaemic stroke patients with subclinical AF detected after stroke. In addition, we address challenging therapeutic decisions in patients with AF and cerebral amyloid angiopathy (CAA) or previous intracerebral haemorrhage (ICH).

### Management of ischaemic stroke patients with pre-existing atrial fibrillation

Up to 32% of patients have AF diagnosed prior to an ischaemic stroke or transient ischaemic attack (TIA) [9]. The Framingham Heart Study identified AF as a strong independent risk factor for stroke, with the risk being five times higher in AF patients compared to those without the arrhythmia [4, 10] The risk of ischaemic stroke in AF patients is influenced by various factors, including the duration of the arrhythmia, [11] the presence of concomitant cardiovascular disease (e.g., arterial hypertension, heart failure), and the patient’s age and can be stratified by the use of prediction tools such as the CHADS_2_ [12], the CHA_2_DS_2_–VASc [13] or the CHA_2_DS_2_–VA score. [14] These scores play a critical role in clinical decision-making regarding anticoagulation therapy for stroke prevention in AF patients. Patients with a score of 2 or greater, which applies to all ischaemic stroke or TIA patients, are considered to be at high risk of stroke and therefore oral anticoagulation (OAC) is generally recommended in American and European AF guidelines [14– 16]. DOACs should be commonly prescribed, since DOACs not only have shown efficacy in reducing the incidence of stroke or systemic embolism, of total, fatal and intracranial bleeding as well as all-cause mortality in AF patients, [17] but also offer advantages such as a more predictable pharmacokinetic profile and require less frequent monitoring in comparison to vitamin K antagonists (VKA). These are only advised in patients with a mechanical valve or moderate to severe mitral stenosis [14].

The evidence for the use of DOACs in severe renal failure is scarce. In line with that, a network meta-analysis of nine studies involving 65,976 patients concluded that in AF patients with advanced CKD, both standard- and reduced-dose apixaban were associated with more favourable outcomes than VKAs, although the certainty of evidence was generally low [18]. Therefore, VKAs are used in cases of severe renal failure (GFR < 15–30 ml/min or where dialysis is required), since DOACs are either contraindicated or approved only under restricted conditions, meaning that VKAs are often used in this situation simply for lack of an alternative.

Regarding the optimal timing of initiation or re-initiation of OAC, the randomised controlled ELAN [19] and OPTIMAS [20] trials examined early initiation of DOACs in ischaemic stroke patients with AF. In ELAN, treatment started within 48 h for minor or moderate stroke, or on day 6–7 for major stroke; in OPTIMAS, within 96 h, with stroke severity as a stratification criterion. Both trials showed that starting early was safe and non-inferior to later, guideline-based initiation, such as the so-called “1-3-6-12-day rule” (starting OAC 1 day after TIA, 3 days after minor stroke, 6 days after moderate stroke, and 12 days after severe stroke) [14]. Importantly, there was no significant increase in haemorrhagic complications. The ELAN and the OPTIMAS trial failed to show a statistically significant reduction in recurrent stroke; only the ELAN trial found a numerically lower number of recurrent strokes during the follow-up period. These findings support earlier DOAC initiation as a viable strategy in this population.

In addition to anticoagulation, current guidelines emphasise the importance of an optimised management of cardiovascular risk factors including treatment of comorbidities such as arterial hypertension, diabetes mellitus, heart failure, obesity and obstructive sleep apnoea. Furthermore, recommendations regarding lifestyle modifications, physical activity, and alcohol intake are made [14]. This should not be neglected in AF patients, especially since, in a meaningful proportion of patients, a second stroke has a different underlying cause than the first one [21, 22].

For patients with any form of AF, rate or rhythm control strategies may be necessary. In a prespecified subgroup analysis of the EAST-AFNET 4 trial in patients with early AF and a history of stroke, early rhythm control significantly reduced the composite cardiovascular outcome compared with usual care (3.7 vs. 7.4 events per 100 person-years; HR 0.52, 95% CI 0.29–0.93) without an increase in safety events, supporting the consistency of the trial’s overall findings in this high-risk subgroup [23]. In line with that, the RAFAS trial showed that early rhythm control initiated within 2 months of an acute ischemic stroke in patients with newly diagnosed AF significantly reduced stroke recurrence and sustained AF at 12 months compared with usual care, without increasing mortality, hospitalizations, or arrhythmia-related adverse events [24]. The ongoing EAST-Stroke trial aims to determine whether early rhythm-control therapy, added to usual care, effectively prevents recurrent stroke and cardiovascular complications in patients with acute ischaemic stroke and AF that has occurred for the first time in the last 12 months [25].

Furthermore, new drug treatment options have yet to prove their value in the management of stroke patients with AF. For example, the OCEANIC-AF phase III trial was halted early after the factor XIa inhibitor asundexian proved markedly inferior to apixaban for stroke/systemic embolism prevention (1.3% vs. 0.4%; HR 3.79), despite fewer major bleeds (0.2% vs. 0.7%) in AF patients with high cardioembolic risk [26]. In contrast, abelacimab, a factor XI monoclonal antibody, reduced bleeding rates by ~ 60–67% versus rivaroxaban in AF patients at moderate-to-high stroke risk in the phase II AZALEA-TIMI 71 trial, prompting phase III testing [27]. The LIBREXIA AF trial, comparing milvexian against apixaban in patients with AF for stroke prevention, is ongoing, with data expected at the end of 2026 [28].

Patients with a history of AF who suffer an ischaemic stroke whilst on adequate anticoagulation, and in whom no other more likely cause of stroke has been identified following a thorough clinical work-up, have suffered what is known as a breakthrough stroke [29]. These patients face a particularly high risk of recurrence and increased mortality [30]. Regarding secondary prevention, current evidence on switching anticoagulants after breakthrough stroke remains inconclusive, with studies comparing maintenance versus switching of OAC showing no clear superiority of either strategy [31, 32]. LAAO has emerged as an alternative therapeutic option, supported by meta-analyses of strokes occurring under anticoagulation, though these analyses do not strictly correspond to the breakthrough stroke definition [33]. The use of LAAO, with or without OAC, is currently being investigated in three randomised controlled trials (ELAPSE [29], LAAOS-4 [34], Occlusion-AF [35]), whilst the INTERCEPT trial [36] is investigating the benefits of bilateral carotid filters in combination with DOACs versus DOACs alone aiming to establish evidence-based management strategies for this high-risk population.

### Management of ischaemic stroke patients without history of atrial fibrillation

**Fig. 1:**
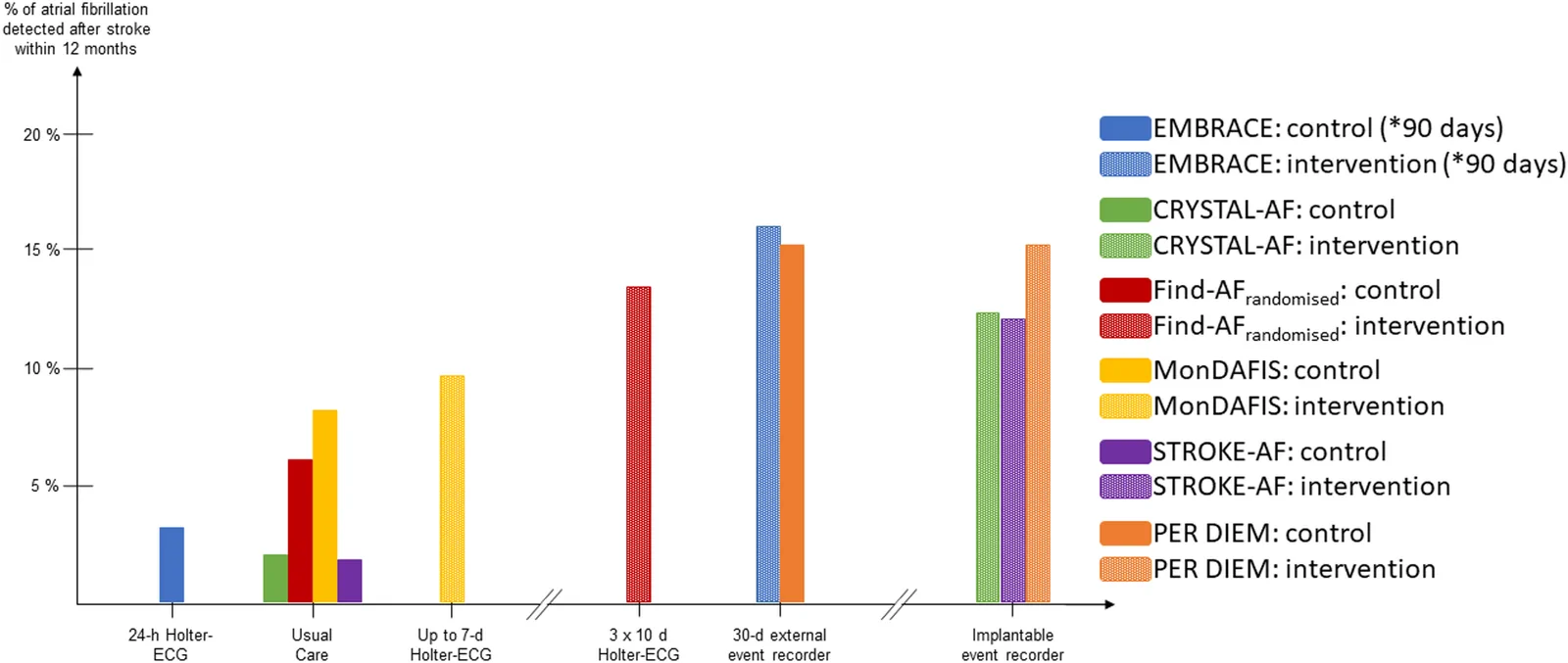
Rates of atrial fibrillation detected within the first 12 months after ischaemic stroke in randomised controlled trials

Each year, up to 1.5 million individuals globally may be newly diagnosed with AF following an ischaemic stroke or TIA [37]. The detection of short and asymptomatic episodes of paroxysmal AF (pAF) can be particularly difficult [23]. Despite differences in inclusion and exclusion criteria (e.g. age, time from index event, stroke aetiology), six randomised controlled trials [38–43], along with a subsequent meta-analysis [44], have demonstrated that extended heart rhythm monitoring following ischaemic stroke or TIA uncovers previously unknown AF in a significantly higher share of patients compared to conventional monitoring regimes (Fig. 1).

However, optimal type and duration of post-stroke heart rhythm monitoring are still being discussed and guideline recommendations vary greatly. In patients with embolic stroke of unknown source (ESUS), the European Society of Cardiology (ESC) guidelines for the diagnosis and management of AF recommend the use of either long-term non-invasive ECG monitors or implantable cardiac monitors for detecting AF.^14^ The European Stroke Organisation (ESO) recommends the initiation of in-hospital ECG monitoring as soon as possible and an outpatient monitoring for more than 48 h, preferably with implantable devices, if feasible, to increase the detection of subclinical AF in patients with cryptogenic stroke [45]. Both the American Heart Association / American Stroke Association as well as the German Society for Neurology recommend prolonged rhythm monitoring, but do not define the type and duration of monitoring in more detail [46, 47]. Other national guidelines propose a continuous ECG monitoring for at least 2 weeks [48] or 7–14 days with an external monitor after embolic stroke [49].

Patients with AF detected after stroke (AFDAS) differ from patients with AF known prior to the stroke. AFDAS is linked to a lower prevalence of vascular risk factors and cardiovascular comorbidities translating into a lower CHA_2_DS_2_–VASc score and a lower AF burden [37, 50–52]. These differences may help explain why previously unknown AF is associated with a lower risk of recurrent ischaemic stroke as compared to patients with AF known before the stroke [37]. Guideline-conform OAC is generally initiated for seofcondary stroke prevention once AFDAS is diagnosed, following standard risk-stratified OAC practice as for known AF, with timing informed by infarct size and haemorrhagic transformation risk [14].

There is broad evidence that prolonged rhythm monitoring leads to increased rates of OAC prescription compared to patients with usual care monitoring and rates of OAC prescription are higher the more intensive and longer the cardiac monitoring was [44].

**Fig. 2:**
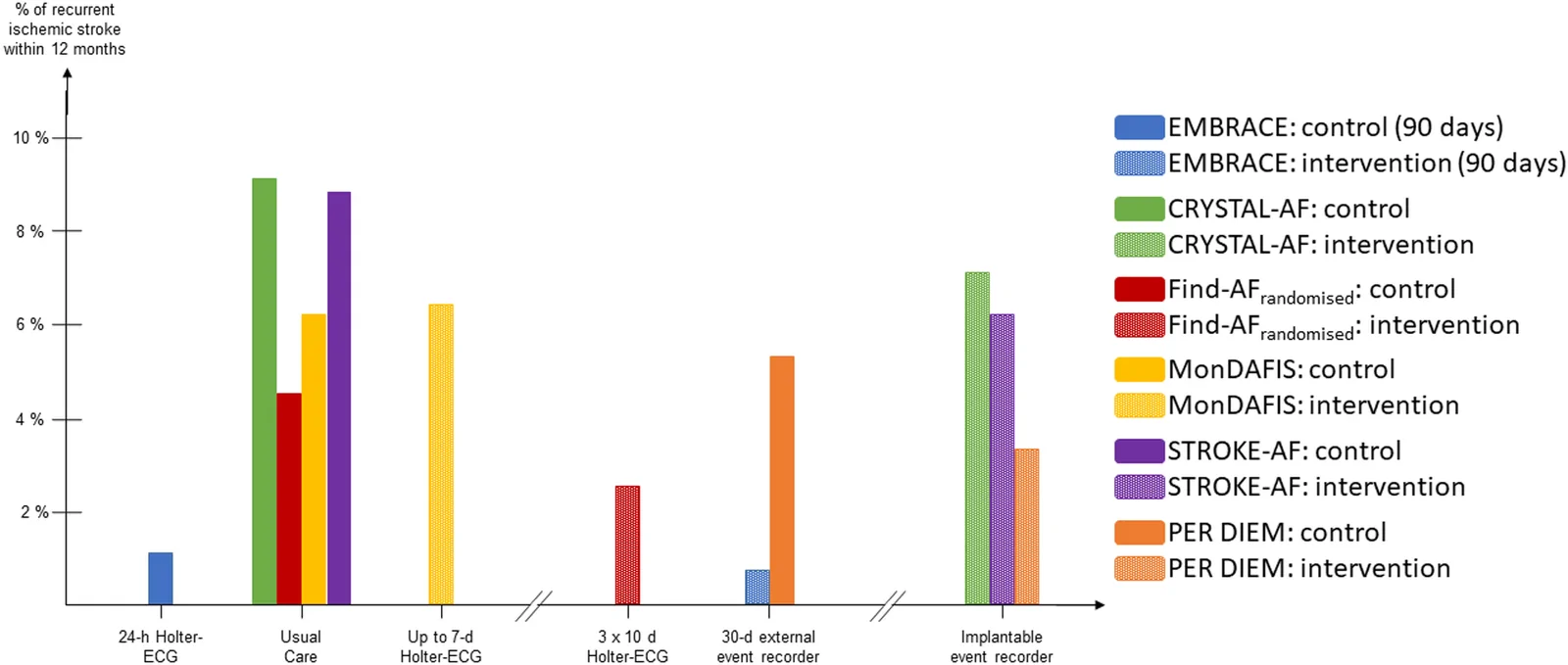
Rates of recurrent ischaemic stroke within the first 12 months after ischaemic stroke in randomised controlled trials

However, whether intensified rhythm monitoring leads to a lower incidence of recurrent stroke through higher rates of OAC prescription in ischaemic stroke patients with newly detected AF after stroke is unclear. Multiple randomised controlled trials [40–42, 53] and observational studies [54–56] have reported a numerical reduction in recurrent ischaemic strokes among patients receiving prolonged monitoring compared to those undergoing standard-of-care monitoring (Fig. 2). The randomised trials were not powered to demonstrate a reduction in recurrent ischaemic stroke, and no statistically significant superiority over standard-of-care monitoring for this clinical outcome has been established.

Two meta-analyses of these studies also found no statistically significant difference in the risk of recurrent stroke between different monitoring approaches [37, 44]. Hence, whether prolonged ECG monitoring after ischemic stroke translates to a clinical benefit by reduction of cardioembolism remains unclear. Two ongoing randomised clinical trials, Find-AF 2 and AF SPICE, investigate this question. The publicly funded Find-AF 2 trial is an investigator-initiated randomised controlled trial of 5,227 patients performed in 52 study centres in Germany and evaluates if a risk-adapted approach of enhanced, prolonged and intensified ECG monitoring to detect underlying paroxysmal AF leads to a reduction in recurrent ischaemic stroke and systemic embolism compared to usual care. [57, 58] Recruitment of participants was successfully concluded in 2024 and results on the primary endpoint are expected for late 2027. AF SPICE is a Swedish multicentre, register-based randomised trial comparing extended (14 + 14-day) versus standard (24–72-hour) ECG monitoring for AF detection with a composite endpoint of recurrent stroke, intracerebral bleeding, and mortality [59]. The recruitment of 2,884 elderly stroke/TIA patients was successfully completed in January 2026.

### Management of ischaemic stroke patients with subclinical AF detected after stroke

**Fig. 3:**
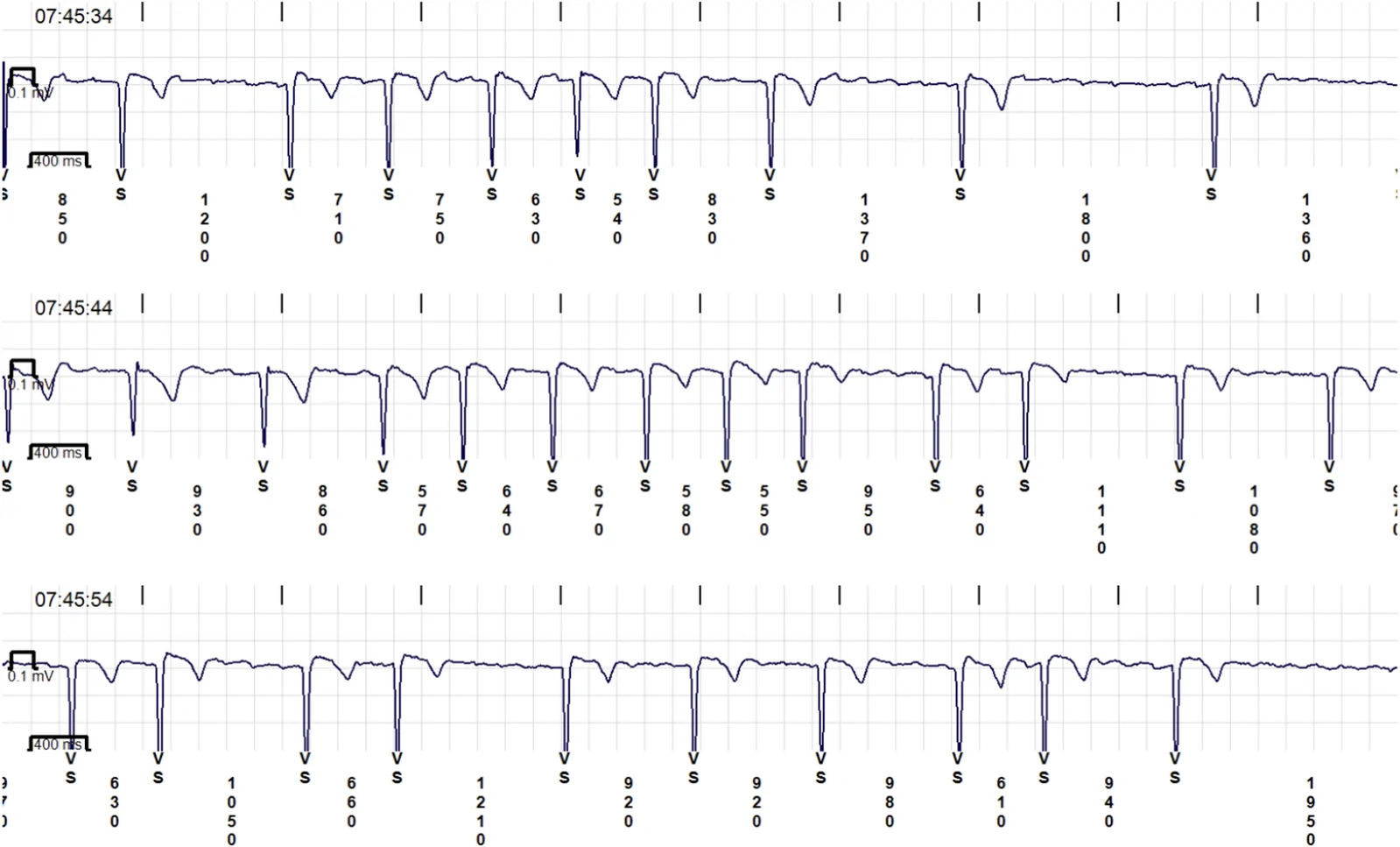
Subclinical atrial fibrillation detected by an implanted cardiac monitor

While clinical AF is readily diagnosed by symptoms and standard electrocardiographic methods, subclinical atrial fibrillation (SCAF), also known as device-detected AF, is defined as episodes of AF that are asymptomatic and are detected only by implantable electronic devices (e.g. pacemakers, implantable cardioverter-defibrillators or cardiac monitors) or wearables and are confirmed by intracardiac electrogram or review of the recorded rhythm on the ECG (Fig. 3) [60, 61].

The increasing use of implantable devices and wearables has substantially improved the identification of arrhythmias that would previously have been undetected using traditional methods like 12-lead or Holter ECG [62].

The ASSERT trial was a milestone in understanding stroke risk in SCAF [60]. A total of 2,580 patients with an implanted cardiac device (mostly pacemakers or ICDs) were followed for the development of SCAF or ischaemic stroke. The first finding was that SCAF was frequent, occuring in 10% in the first three months and > 50% after four years of follow-up. The second important finding was that in the first three months the risk of ischaemic stroke or systemic embolism was 2.5 times higher in patients with SCAF compared with those without SCAF. The third important observation was that the ischaemic stroke risk was only increased for SCAF with a duration of at least 24 h, but not in those with shorter SCAF episodes [11].

Two recent randomised controlled trials, the ARTESiA and the NOAH-AFNET 6 trial, have evaluated the efficacy of DOACs in people with implantable devices, but only approximately 10% of patients had a history of TIA or ischaemic stroke [63, 64]. Although annual stroke rates (0.8–1.2%/year and 0.9–1.1%/year, respectively) were low in patients with SCAF, ARTESiA showed a risk reduction for stroke or systemic embolism with apixaban compared with aspirin in the overall study cohort. However, an increased rate of major bleeding events was observed. In contrast, the NOAH-AFNET 6 trial did not demonstrate a statistically significant reduction in stroke and reported increased bleeding, leading to early study termination [64]. Two recent meta-analyses of both trials indicated that OAC with edoxaban or apixaban reduces the risk of stroke in patients with SCAF, but increases the risk of major bleeding, leaving open questions on the net clinical benefit of OAC for SCAF in this mixed population [65, 66].

Data reporting the incidence of SCAF among stroke/TIA patients are sparse. In a multicentre cohort study of 789 patients with cryptogenic stroke and an insertable cardiac monitor, SCAF was detected in 29.0% of patients during a mean follow-up of 27.3 months, with a cumulative detection rate of 37.0% at 48 months after the index stroke [67]. In a subgroup analysis of the ARTESiA trial among patients with SCAF and a prior stroke or TIA, apixaban lowered the 3.5-year risk of stroke or systemic embolism by 7%, whereas the risk reduction was only 1% in those without a prior cerebral ischaemic event, demonstrating that these patients derive substantially greater absolute benefit from apixaban than those without prior cerebrovascular events. This greater efficacy was partly counterbalanced by a higher bleeding risk: major bleeding occured in 3% in the stroke/TIA subgroup versus 1% in patients without [68]. As a result, contemporary expert consensus statements recommend individualised management, incorporating risk stratification tools, such as the CHA₂DS₂-VA^14^ and HAS-BLED scores [69], arrhythmia burden, overall comorbidity profile, and patient preferences in OAC decisions for patients with SCAF [61, 70, 71]. In patients with a high risk of thromboembolism, especially in patients with a history of TIA or ischaemic stroke or notable AF burden > 24 h, OAC may be considered after weighing the elevated bleeding risk. In our opinion, in all other patients, the risk of ischaemic stroke is too low and the risk reduction by anticoagulation too small (or absent) to give a recommendation for anticoagulation. In these lower-risk patients, conservative management with close follow-up may be sufficient.

### Risk of ischaemic stroke in different atrial fibrillation patient groups

**Fig. 4:**
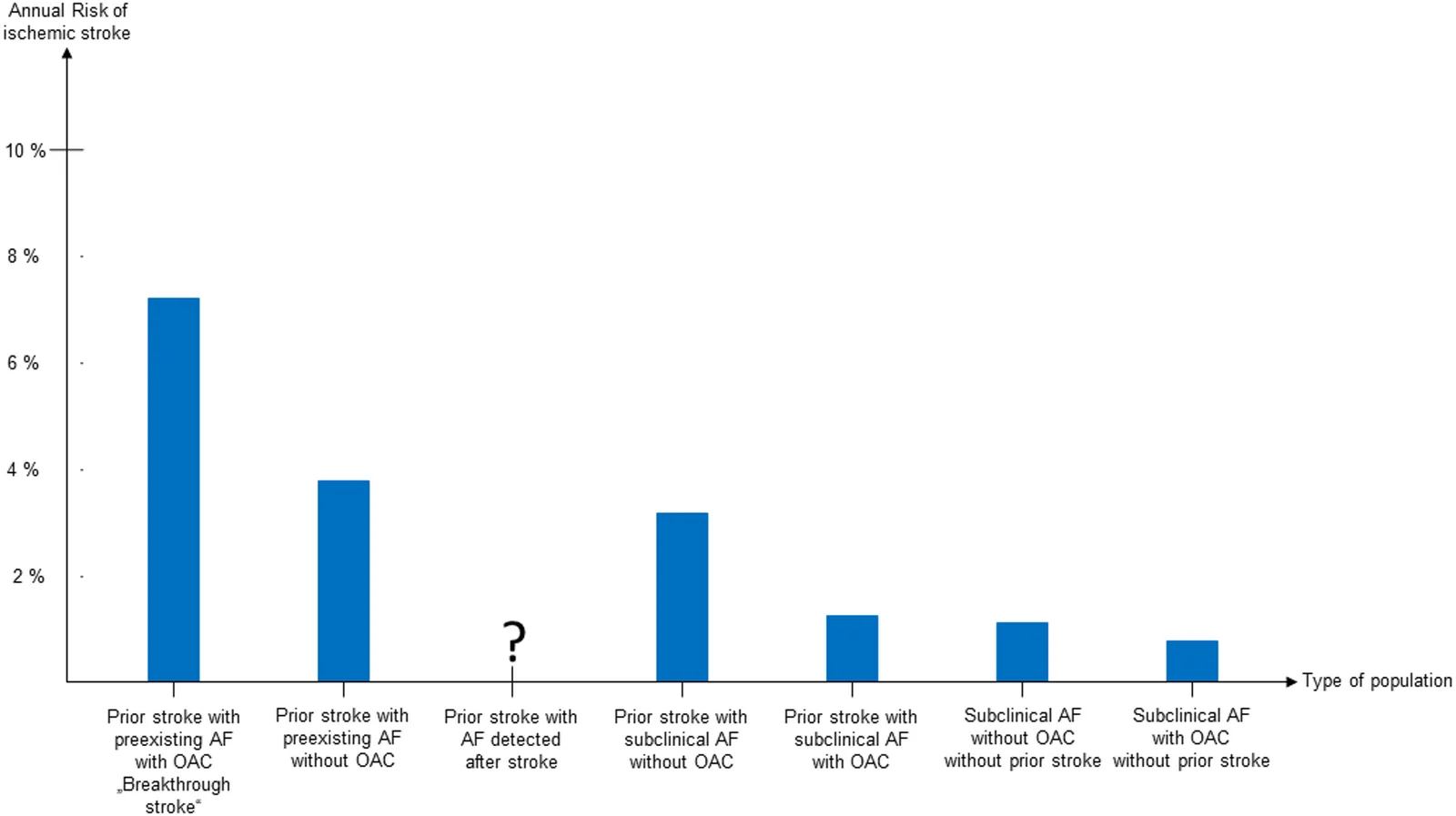
Annual ischaemic stroke risk in various atrial fibrillation populations

Apart from conventional tools such as the CHA_2_DS_2_–VA(Sc) score, the risk of ischaemic stroke in patients with AF is primarily determined by three factors: (a) clinical versus subclinical AF, (b) a history of TIA or ischaemic stroke, and (c) the presence or absence of OAC. It is lowest in patients with SCAF without prior stroke and with anticoagulation and increases stepwise with the highest risk in patients with clinical AF, a history of ischaemic stroke and no anticoagulation (see Fig. 4) [30, 68].

#### Challenging therapeutic situations in neurological patients with atrial fibrillation

#### Patients with cerebral amyloid angiopathy and atrial fibrillation

**Fig. 5:**
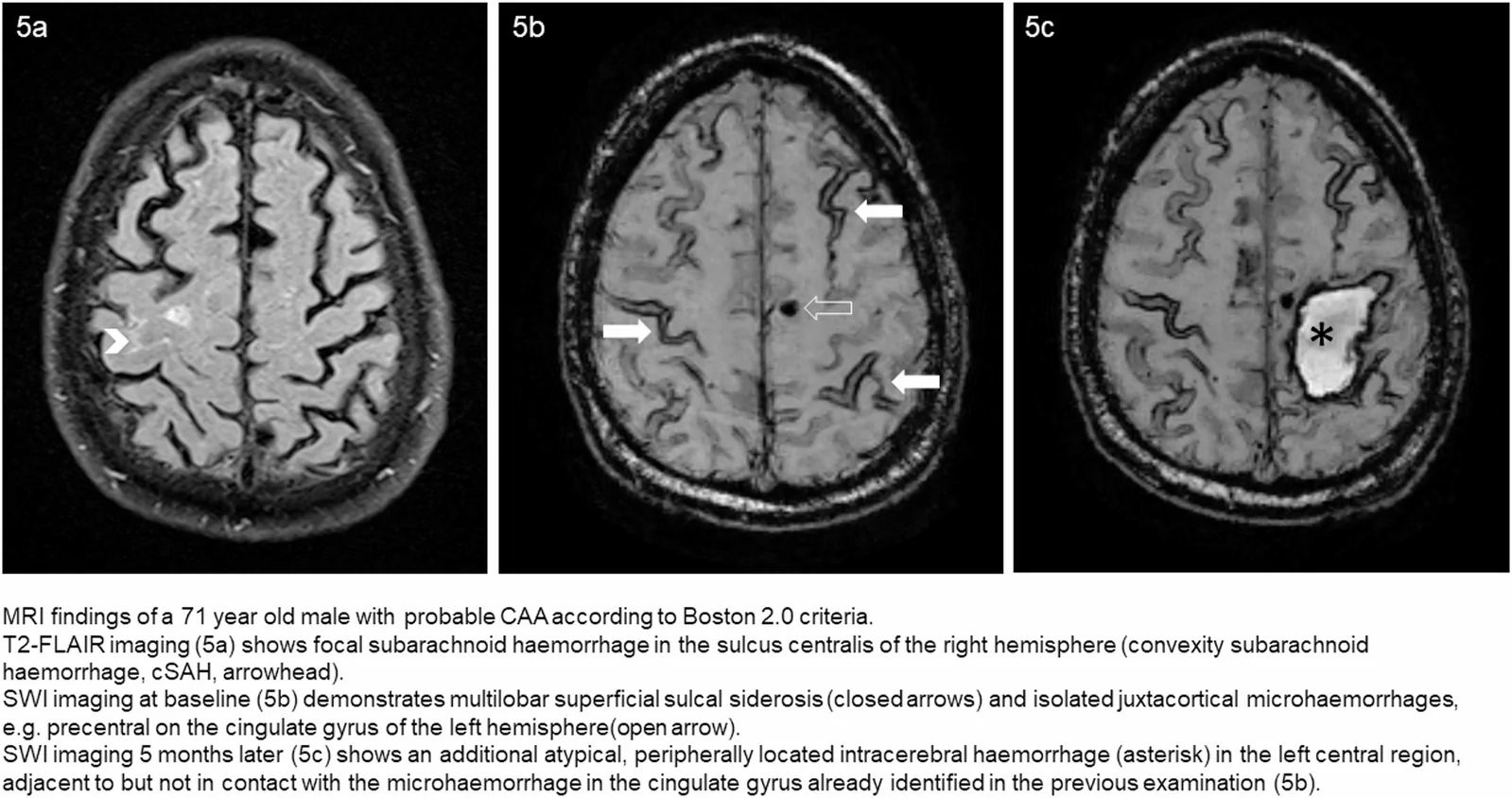
Classic radiological features of cerebral amyloid angiopathy

Cerebral amyloid angiopathy (CAA) is an age-related cerebrovascular disorder, which typically presents with one of three clinical patterns: (a) acute lobar intracerebral haemorrhage sparing deep grey-matter structures, (b) gradually progressive cognitive decline affecting mainly processing speed and executive function, or (c) recurrent, stereotyped transient focal neurologic spells (lasting < 30 min) that spread along contiguous body regions [72]. Its pathophysiology is marked by the deposition of amyloid beta-peptide in cerebral arterioles and capillaries [73]. This accumulation leads to increased vascular fragility, making vessels more susceptible to haemorrhage. Cerebral imaging therefore shows intracerebral lobar hematomas, cerebral microbleeds, focal subarachnoid haemorrhages and cortical superficial siderosis (Fig. 5) [74, 75].

Imaging features of CAA and AF frequently coexist in the elderly, presenting a complex and challenging clinical scenario [76]. While OAC is effective in reducing ischaemic stroke risk in AF patients [77], its use in those with concomitant CAA raises concerns due to the risk of haemorrhagic strokes, necessitating a careful balance between preventing ischaemic events and avoiding haemorrhagic complications.

In patients with competing risks of thromboembolic as well as bleeding events, the CHA₂DS₂-VA(Sc) score [13, 14] as well as the HAS-BLED [69] score are usually used to determine the patient’s individual risk constellation. However, the CHA₂DS₂-VA(Sc) and HAS-BLED scores were derived and validated in general AF populations and do not account for CAA-specific pathology; consequently, HAS-BLED has been shown to systematically underestimate bleeding risk in patients with CAA [78]. Critically, neither score incorporates neuroimaging markers such as cerebral microbleeds or cortical superficial siderosis, which are the strongest predictors of ICH in this population,^75^ resulting in a substantial mismatch between predicted and observed risk. The decision to initiate OAC must, therefore, consider the severity of CAA as well as the patient’s overall haemorrhagic risk profile. For this purpose, both the patient’s history and clinical as well as MRI findings should be used.

In a recent retrospective, propensity-score-matched cohort study in 574 elderly patients with a database diagnosis of CAA and newly diagnosed high-risk AF, but without a history of ischemic or haemorrhagic stroke, the use of apixaban or rivaroxaban was associated with a significantly lower 3-year risk of ischemic stroke (1.0% vs. 5.7%, *p* = 0.016) and lower mortality (27.9% vs. 33.5%, *p* = 0.049), without a significant increase in intracerebral haemorrhage (3.0% vs. 3.9%, *p* = 0.21) or major haemorrhage (8.1% vs. 10.7%, *p* = 0.09). These findings suggest that OAC may be safe and beneficial in this specific subgroup of CAA and AF patients who have not yet experienced a cerebral haemorrhagic event, though the retrospective design and use of diagnostic codes rather than MRI-based CAA criteria warrant cautious interpretation [79]. However, the risk of ICH is particularly high in CAA patients with previous ICH, presence of disseminated cortical superficial siderosis and / or a higher number of cerebral microbleeds. [80] Furthermore, longer duration of OAC and OAC with VKA increases bleeding risk. [73, 76] While bleeding risk is increased, CAA is also associated with an increased risk for ischaemic infarctions, especially microangiopathic infarcts. [81] The thromboembolic risk is further increased in CAA patients with concomitant hypercoagulopathy (including paraneoplastic coagulation disorders), mechanical valves, atrial or ventricular thrombus and previous ischaemic stroke [82].

After an individualised risk-benefit analysis, we propose shared decision-making, engaging patients in discussions about the potential benefits and risks. If the decision is in favour of OAC, DOACs should be preferred, since they have demonstrated a lower risk of ICH compared to VKA in the general AF population [82]. However, their safety in patients with CAA remains uncertain. Some studies suggest that certain DOACs may have a more favourable profile, but robust evidence is lacking, and the choice of anticoagulant should be individualised. Under DOAC therapy, an ongoing evaluation of neurological status and periodic imaging may help detect early signs of haemorrhage, allowing for timely adjustments in therapy.

Alternatively, patients at higher risk of bleeding may be offered a left atrial appendage occluder (LAAO), which offers a non-pharmacological alternative to reduce stroke risk without the need for long-term OAC. In three randomized trials of LAAO versus OAC in patients with AF, CHAMPION-AF showed in 3000 patients LAAO to be non-inferior to DOAC therapy for the composite of cardiovascular death, stroke, or systemic embolism at 3 years, with significantly less non-procedure-related bleeding [83], whereas in a post-ablation cohort of 1600 patients the OPTION trial met non-inferiority for the primary efficacy endpoint and demonstrated superiority for major bleeding with LAAO [84]. In contrast, CLOSURE-AF, conducted in 912 patients at high combined risk of stroke and bleeding, failed to demonstrate non-inferiority of LAAO compared with physician-directed medical therapy (predominantly DOACs), [85] suggesting that the benefit of LAAO over anticoagulation may be population-dependent. In three observational cohort studies of patients with CAA (with or without ICH) and AF a LAAO was found to be a safe and tolerable procedure to reduce stroke risk [86–88].

In summary, the management of OAC in patients with both AF and CAA remains challenging due to the competing risks of ischaemic and haemorrhagic strokes. A patient-centred approach is essential, incorporating thorough risk assessments, consideration of LAAO, and continuous monitoring to optimise outcomes in this complex patient population.

It is possible, however, that disease-modifying approaches that reduce amyloid-β production or enhance its clearance , which are currently still at the experimental stage, may influence treatment decisions in the future. [89].

#### Patients with previous intracranial haemorrhage and atrial fibrillation 

More than 3 million people worldwide experience an intracranial haemorrhage (ICH) every year [90]. Around 40% of patients suffering from ICH die within the first month and more than half of those who survive require assistance from others for daily activities [91] Among ICH survivors, AF is present in 10–35% of patients, and AF-related anticoagulation contributes to approximately 20% of all ICHs [92, 93].

The annual recurrence risk of ICH is estimated to range from 1.8% to 7.4% [94] and is even higher in patients using antithrombotic and OAC therapy [95]. the increased risk of bleeding, ICH patients also have an elevated risk of ischaemic events of up to 20% wthin 5 years after ICH [96]. In ICH patients with comorbid AF, the risk of ischaemic stroke is significantly increased compared with those without AF (6.3 versus 0.7 per 100 patient-years) [97].

Hence, effective secondary prevention for survivors of ICH with coexisting AF remains a clinical challenge, as these patients face an elevated risk of both recurrent ICH and ischaemic stroke.

SoSTART, [98] APACHE-AF, [99] NASPAF-ICH [100], PRESTIGE-AF [101] and the recently presented ENRICH-AF (NCT: 03950076) are randomised controlled trials that investigated whether initiating or resuming OAC is superior to avoiding anticoagulation (no antithrombotic treatment or antiplatelet therapy) in survivors of ICH with concomitant AF. An overview of the aforementioned completed trials is given in Tables 1 and 2 shows key data on the currently ongoing study ASPIRE.

**Table 1:** Overview of completed randomised clinical trials investigating oral anticoagulation versus no anticoagulation in patients with intracranial haemorrhage and atrial fibrillation

Trial	*n* intervention group	*n* control group	Median age (IQR) [years]	female sex [%]	rate of lobar ICH [%]	CHA₂DS₂-VASc Score [Median (IQR)]	Procedure intervention group	Procedure control group	Median Follow-up (IQR) [years]	recurrent ICH intervention group [%]	recurrent ICH control group [%]	ischemic stroke intervention group [%]	ischemic stroke control group [%]
**SoSTART** [75]	101	102	79 (74–85)	37	36	4 (3–5)	DOAC or vitamin K antagonist	31% APT, 69% no APT	1.2 (1.0–2.0)	8	4	3	19
**APACHE-AF** [76]	50	51	77 (70–83)	40	28	4 (3–5)	Apixaban	51% APT, 49% no APT	1.9 (1.0-3.1)	8	2	12	12
**NASPAF-ICH** [77]	21	9	76 (68–82)	44	27	not documented	DOAC	ASS 81 mg/d	Mean: 1.5 (SD 0.5)	0	0	0	11
**PRESTIGE-AF** [78]	158	161	79 (73–83)	35	30	4 (3–6)	DOAC	33% APT, 67% no APT	1.4 (0.7–2.3)	9	1,2	0,6	12,4
**ENRICH-AF**	467	477	Mean 77 (SD 9)	39		4 (3–5)	Edoxaban	52% APT, 48% no APT	13 (6–21) months	6.6	2.9	6.2	10.3

IQR: Interquartile range, DOAC: direct oral anticoagulant, APT: anti platelet therapy, SD: standard deviation

**Table 2:** Overview of the ongoing randomised clinical trial ASPIRE investigating oral anticoagulation versus no anticoagulation in patients with intracranial haemorrhage and atrial fibrillation

Ongoing Trial	NCT	Sample size	Procedure intervention group	Procedure control group	Primary endpoint	Status
**ASPIRE**	03907046	700 planned	Apixaban	Aspirin 81 mg/d	stroke or death	recruiting

Recent meta-analyses of the abovementioned randomised controlled trials and several observational trials, partly including more than 9,000 patients, consistently demonstrate that resuming DOAC in survivors of ICH with AF significantly reduces the risk of ischaemic stroke and thromboembolic events compared with no anticoagulation, but this benefit is counterbalanced by a significantly elevated risk of recurrent intracerebral haemorrhage, with no clear difference in short-term mortality [102– 106]or “net clinical benefit” (combining first incidence of ischaemic stroke and first occurence of recurrent ICH) [104].

Risk stratification may therefore be needed in order to identify patients with ICH and AF benefiting from OAC. One approach for risk stratification is the haematoma location (lobar versus non-lobar). Observational studies have indicated that lobar haemorrhages carry a 2- to 3-fold increased risk of recurrent ICH compared with non-lobar haematomas [94, 97] Furthermore, an interim safety analysis of the ongoing ENRICH-AF trial (NCT03950076) revealed a markedly heightened risk of recurrent ICH in anticoagulated patients with lobar ICH, leading the steering committee to stop edoxaban in patients with lobar ICH and halted further enrolment of such patients. In contrast, in post-hoc analyses of the PRESTIGE-AF trial, the rate of recurrent ICH did not appear to be higher in patients with lobar compared to non-lobar location of the index ICH. But these results should be interpreted with caution, since the number of recurrent ICH was very small [107].

Future options for ICH patients with coexisting AF could be new approaches, such as rhythm control or LAA occlusion. For the latter, there is limited evidence from observational studies, which showed LAAO to be an effective and feasible alternative in patients with previous ICH and AF [108] The CLEARANCE trial (NCT04298723) is currently investigating LAA occlusion in patients with AF and a history of ICH.

In summary, the decision on whether patients with ICH and AF should receive OAC remains challenging, given the persisting uncertainty regarding the optimal balance between ischaemic and haemorrhagic risk. Current evidence supports individualised, multidisciplinary decision-making that addresses modifiable bleeding risk factors, such as achieving optimal blood pressure control, and accounts for imaging parameters over time (e.g., lobar haemorrhage, associated CAA), together with shared decision-making with patients, while adequately powered trials are still needed to refine patient selection, timing, and anticoagulant choice.

## Conclusions

AF remains a leading, modifiable risk factor for ischaemic stroke, yet its optimal management differs substantially across clinical scenarios (pre-existing AF, newly detected clinical AF, and subclinical AF after ischaemic stroke), requiring tailored diagnostic and therapeutic strategies. While DOACs represent the cornerstone of stroke prevention, their use in complex populations such as ICH survivors or patients with cerebral amyloid angiopathy demands careful, individualised risk-benefit assessment incorporating imaging, comorbidities, and patient preferences. Emerging approaches, including left atrial appendage occlusion, carotid artery filter systems, factor XIa inhibitors or rhythm control may refine future management. Ultimately, interdisciplinary collaboration and shared decision-making remain essential to balance thromboembolic and haemorrhagic risks in this heterogeneous, high-risk patient population.

## Data Availability

No datasets were generated or analysed during the current study.

## Abbreviations

AF: Atrial fibrillation
AFDAS: Atrial fibrillation detected after stroke
AF SPICE: Atrial Fibrillation Screening Post Ischemic Cerebrovascular Events
APACHE-AF: Randomised controlled trial: Apixaban versus Antiplatelet Drugs or No Antithrombotic Drugs after Intracerebral Haemorrhage in Patients With Atrial Fibrillation
APT: anti platelet therapy
ARTESiA: Randomised controlled trial: Apixaban for the Reduction of Thrombo-Embolism in Patients With Device-Detected Sub-Clinical Atrial Fibrillation
ASPIRE: Ongoing randomised controlled trial: Anticoagulation in ICH Survivors for Stroke Prevention and Recovery
ASSERT: Randomised controlled trial: Asymptomatic Atrial Fibrillation and Stroke Evaluation in Pacemaker Patients and the Atrial Fibrillation Reduction Atrial Pacing Trial
CAA: Cerebral amyloid angiopathy
CHADS₂: Congestive heart failure, Hypertension, Age ≥ 75, Diabetes mellitus, prior Stroke/TIA/thromboembolism (doubled), stroke risk score
CHA₂DS₂-VA: CHA₂DS₂-VASc score without the sex category (female) component
CHA₂DS₂-VASc: Congestive heart failure, Hypertension, Age ≥ 75 (doubled), Diabetes mellitus, prior Stroke/TIA/thromboembolism (doubled), Vascular disease, Age 65–74, Sex category (female), stroke risk score
CLEARANCE: Randomised controlled trial: Comparison of LAA-Closure vs. Oral Anticoagulation in Patients With NVAF and Status Post Intracranial Bleeding
DOAC(s): Direct oral anticoagulant(s)
EAST-STROKE: Randomised controlled trial: Early treatment of Atrial fibrillation for Stroke prevention Trial in acute STROKE
ENRICH-AF: Randomised controlled trial: Edoxaban versus No Antithrombotic Treatment in Patients With Previous Intracerebral Haemorrhage and Atrial Fibrillation
ESC: European Society of Cardiology
ESO: European Stroke Organisation
ESUS: Embolic stroke of undetermined source
HAS-BLED: Hypertension, Abnormal renal/liver function, Stroke, Bleeding history/predisposition, Labile INR, Elderly, Drugs/alcohol concomitantly — bleeding risk score
ICH: Intracerebral haemorrhage
ICD: Implantable cardioverter-defibrillator
IQR: Interquartile range
LAA: Left atrial appendage
LAAO: Left atrial appendage occlusion
MRI: Magnetic resonance imaging
NASPAF-ICH: Randomised controlled trial: Non-vitamin K Antagonist Oral Anticoagulants or Aspirin for Patients With Atrial Fibrillation and Previous Intracerebral Haemorrhage
NOAH-AFNET 6: Randomised controlled trial: Non-vitamin K Antagonist Oral Anticoagulants in Patients With Atrial High Rate Episodes
NCT: National Clinical Trial (identifier, ClinicalTrials.gov)
OAC: Oral anticoagulation / oral anticoagulant
SCAF: Subclinical atrial fibrillation
SD: Standard deviation
SoSTART: Randomised controlled trial: Start or Stop Anticoagulants Randomised Trial
PRESTIGE-AF: Randomised controlled trial: Direct Oral Anticoagulants versus No Anticoagulation for the Prevention of Stroke in Survivors of Intracerebral Haemorrhage with Atrial Fibrillation
TIA: Transient ischaemic attack
VKA: Vitamin K antagonists
